# Differential Effects of Dietary Oregano Essential Oil on the Inflammation Related Gene Expression in Peripheral Blood Mononuclear Cells From Outdoor and Indoor Reared Pigs

**DOI:** 10.3389/fvets.2021.602811

**Published:** 2021-02-25

**Authors:** Katia Cappelli, Marcella Sabino, Massimo Trabalza-Marinucci, Gabriele Acuti, Stefano Capomaccio, Luigi Menghini, Andrea Verini-Supplizi

**Affiliations:** ^1^Dipartimento di Medicina Veterinaria, University of Perugia, Perugia, Italy; ^2^Dipartimento di Farmacia, University of Chieti, Chieti, Italy

**Keywords:** immune response to environment, gene expression, oregano essential oil, pig, rearing system, PBMC, inflammation

## Abstract

Intensive farming systems represent a stressful environment for pigs and negatively influence neuroendocrine functions, behavior, and performance. Outdoor farming is an alternative option, which is thought to imply several beneficial effects for the animal. Dietary essential oils are known to be an innovative strategy to improve pig health and performance, and oregano essential oil (ORE) possesses beneficial effects due to its antimicrobial, anti-fungal, and antioxidant properties. We tested the effect of dietary ORE on peripheral blood mononuclear cells (PBMCs) in 36 growing pigs, either reared under indoor or outdoor conditions. Quantitative real-time PCR (RT-qPCR) assay was used to evaluate the effect of diet (control vs. ORE) and the time of sampling (T1−120 days vs. T2−190 days) on the expression of inflammatory and immune-related genes (*TNF, IL1*β, *IL8, IL18, IL10, IL1RN, STAT3, HSP90, ICAM-1*, and *NFKB1*). Under outdoor condition, the majority of transcripts were upregulated (*p* < 0.05), assuming a general inflammatory status (TNF, HSP90, NFKB1, IL1β, and STAT3). However, an interaction between diet and the farming system was observed: *HSP90, NFKB1*, and *STAT3* were downregulated (*p* < 0.05) in the outdoor reared pigs when fed the ORE diet. Our study showed that bioactive compounds of ORE exert their activity, especially when the animals are exposed to stressful stimuli. Dietary ORE can be an acceptable strategy to help pigs tolerate the stress related to the harsh, outdoor, rearing conditions.

## Introduction

The increase in demand of meat production has led to a worldwide increase in intensive farming systems, which usually represent a stressful environment for the animals (1). In intensively reared pigs farms, high temperature, small space, and noise are some of the stress factors which negatively affect animal growth and meat quality (2). Prolonged stressful stimuli influence the immune system, altering leukocyte activation and the release of immunoglobulin, cytokines, and inflammatory factors (3–5).

The outdoor farming system is generally considered a better strategy to improve animal welfare and health compared to the intensive indoor farming, even though beneficial and stressful stimuli in both rearing systems can influence performance (2, 6–11). With increased space being available and the more natural environment in outdoor farming representing advantages to improve pig welfare (12, 13), the harsh environmental conditions can negatively influence the performance of the animals (6, 7, 14, 15). Pigs in the outdoor rearing system, indeed, are exposed to environmental stressors (i.e., extreme climatic events, physical exercise, and predators), which can negatively affect their immune system and increase their susceptibility to diseases (16–18). Lymphocyte proliferation and natural killer cell activity can be impaired by acute exposure to thermal stress (19–21) and, in addition to the environmental stressor, exercise exacerbates the reaction of the immune system and activates pro-inflammatory and anti-inflammatory pathways, depending on the intensity and duration of stress (22, 23).

Moreover, the genetic background of current pig breeds, which have been selected to optimize the performance in indoor breeding systems, probably compounds the negative effects of the outdoor rearing systems. Thus, many management practices need to be reassessed when certain genotypes are recruited (24).

Among the possible management interventions, the incorporation of plant-derived compounds into the diet appears to be a strategy to improve swine health, performance, and food-derived quality (25, 26). Essential oils, from plant derivatives, are used for their antimicrobial, antioxidant, and anti-inflammatory properties (27–30). Among essential oils, oregano essential oil (ORE) is used in the pig industry for its beneficial effects, ranging from improved performance to increased pork quality (31, 32). It has been shown that oregano has anti-inflammatory and antimicrobial effects (33–35), by modulating the cytokine levels and immunity-related transcription factors (36, 37). In addition, oregano stimulates digestion with effects on enterocytes, by accelerating their renewal rate, improving their capacity for nutrient absorption, and reducing pathogen contamination (38).

In a previous study, we have observed that outdoor rearing conditions have a major, negative impact on the growth rate of pigs and that dietary ORE is effective in reducing these performance losses due to the rearing system by increasing the oxidative status of the animals (11).

In this study, we hypothesized that these environmental factors may influence the expression of inflammatory and immune response-related genes in the peripheral blood mononuclear cells (PBMCs) of growing pigs, either under indoor or outdoor conditions, and that the dietary supplementation with ORE can affect this response. The PBMC population has already been chosen to correctly monitor the immune response in pigs (39, 40) and the effect of the rearing system on this response (3–5).

## Materials and Methods

### Housing, Animals, and Dietary Treatments

The Department of Pathology, Diagnostics and Veterinary Clinics of the University of Perugia approved the experimental project, and all procedures were in accordance with the European recommendations (European Parliament and the Council of the European Union, 2010) for the protection of animals used for scientific purposes.

The experiment was conducted from June 2012 to January 2013. A total of 36 male Suffolk® hybrid pigs, with an average live weight (LW) of 41.87 ± 1.23 kg, were allowed 35 days to adapt to the experimental conditions prior to the commencement of the study.

Animals were then balanced for LW and litter and blocked into four groups according to a 2 × 2 factorial design: two rearing systems, (a) Outdoor (OUT): ~280 m^2^/pig in outdoor pens provided with huts for shelter and (b) Indoor (IN): 2 m^2^/pig in a building with natural ventilation and wheat straw for bedding), and two dietary treatments. The two diets, formulated to be isonitrogenous and isoenergetic and to meet the nutrient requirements of National Research Council (NRC) for growing pigs (41), were as follows: (a) a commercial pelleted feed (CTRL) and (b) a CTRL with 0.2% (as fed) ORE (*Origanum vulgare* L.) adsorbed on inert adsorbents (ORE).

The experimental diets were administered for 190 days until slaughter; each of them consisted of two feeds administered in two consecutive phases: early finisher (up to 100 kg LW) and late finisher (100 kg LW to slaughter). During the first 35 days of the experiment (adaptation period—T0), all groups of pigs were fed the CTRL diet. More details concerning the composition of the diets, the housing conditions, and the climatic details recorded during the experiment are described in Forte et al. (11).

Both outdoor and indoor pigs were reared in a farm located in Umbria region, Central Italy. The climatic classification of the region according to the Köppen-Geiger system (42) is Cfa (warm temperate climate, fully humid, with hot summer) (11). The natural photoperiod (43°110 northern latitude and 12°610 eastern longitude) was maintained for the whole experimental period, and the main registered meteorological variables were as follows: maximum and minimum temperatures in July and January were 40.6–13.2 °C and 16.1–3.5 °C, respectively; the relative humidity ranged from 55.5 to 90.3%; the mean wind speed at the animal level in July and January ranged from 9 to 39 km/h and from 6 to 41 km/h, respectively; the average rainfall was 48 mm in July and 120 mm in December (average rainfall per year: 901 mm).

### Phytochemical Characterization of the ORE

A commercial sample of ORE in the form of white powder was used as a raw material. A commercial sample of ORE, adsorbed on calcium carbonate, calcium aluminum silicate, and potassium aluminum silicate (OR200 Greenvet, Apa-CT, Forlì, Italy), was formulated to be used in animal feeding as a feed supplement. An aliquot of powder (2 g) was transferred in a conic vial and subjected to ultrasound-assisted extraction for 30 min with 25 ml of methanol 70% in water. Afterward, the sample was centrifuged (5,000 g, 10 min), and the residue was diluted in fresh solvent and was extracted for a second time. After centrifugation, the two liquids were combined, taken to final volume, and directly used for phytochemical investigation in terms of total phenols (by Folin-Cicalteau method), total flavonoids (AlCl3 method), total proanthocyanidins (p-dimethylaminocinnamaldehyde assays), and total antioxidant capacity [2,2′-azino-bis(3-ethylbenzothiazoline-6-sulfonic acid) (ABTS) test]. The detailed procedures for each method are described in previously published paper (43).

For quantitative determination of the carvacrol content, the extract was filtered (0.45 μm pore filter) and injected in an analytical HPLC-PDA system according to Skendi et al. (44) with a slight modification. Briefly, the chromatographic system consisted of a binary system pump (Jasco PU-2080, Tokyo, Japan) and a diode array detector (Jasco MD-2010, Tokyo, Japan) equipped with a reversed-phase Kinetex C18 column (250 × 4.5 mm, particle size; Phenomenex, Torrance, USA). The mobile phase consisted of 0.1% acetic acid (A) and methanol (B) using a gradient elution from 70% A to 35% A in 50 min. The flow rate was 1 ml/min. Peak identification was performed by a direct comparison of retention times and UV–vis spectra with carvacrol pure standard (Sigma Aldrich, St. Louis, MO, USA). Quantification was performed by the comparison with a calibration curve (R^2^ = 0.998). Analyses were performed in triplicate. Results of the phytochemical profile are reported in Table 1.

**Table 1:** Phytochemical profile.

**Items**	**Results**
Total phenolic content (mg GAE/g)	75.7 ± 3.3
Total flavonoid content (mg RE/g)	0.57 ± 0.2
Total proanthocyanidins (mg CE/g)	45.17 ± 2.2
ABTS (mg TE/g)	3.31 ± 0.4
Carvacrol (mg/g)	6.06 ± 0.8

*Values are reported as mean ± standard deviation; GAE, gallic acid equivalent; RE, rutin equivalent; CE, catechin equivalents; TE, Trolox equivalent*.

### Sample Collection, RNA Extraction, and Reverse Transcription

Blood samples were drawn from the jugular vein of all animals at the three time-points after the adaptation period of 35 days (T0), at 120 days (T1), and at the end of the experiment (T2, 190 d), and peripheral blood mononuclear cells (PBMCs) were isolated with gradient centrifugation of Ficoll-Paque™ PLUS Media (Cytiva, Marlborough, MA, USA) according to the manufacturer's instructions. In brief, we diluted total blood with a phosphate-buffered saline (PBS) medium 1:2 and then stratified on Ficoll-Paque gradient 1:4 centrifugation. After centrifugation, we withdrew the PBMC rings that were washed twice in the PBS solution.

Total RNA was extracted from PBMCs using the Aurum Total RNA Fatty and Fibrous Tissue Kit (Bio-Rad, Herculers CA, USA) according to the manufacturer's instructions, and treatment with the Ambion TURBO DNA-*free* kit (ThermoFisher Scientific, Waltham, MA, USA) was carried out, to avoid DNA contamination.

RNA quantity and integrity were evaluated with the Quant-iT RNA Assay Kit (Invitrogen, Doret, UK) in a VersaFluor fluorometer (Bio-Rad, Herculers CA, USA) and denaturing agarose gel electrophoresis, respectively. Purified RNA (500 ng) was reverse transcribed using random hexamers and the Superscript III Reverse Transcriptase (Invitrogen, Dorset, UK), according to the manufacturer's instructions. Successful reverse transcription was confirmed by PCR amplification of the Sus scrofa β-actin gene (XM_003357928).

### Primers Design, Reference Genes (RGs) Selection, and Quantitative Real-Time PCR (RT-qPCR)

Primers were designed using the Primer-BLAST software (45) for six RGs: β-actin (*ACTB*), glyceraldehyde-3P-dehydrogenase (*GAPDH*), hypoxanthine ribosyltransferase (*HPRT*), β-2-microglobin (*B2M*), succinate dehydrogenase complex subunit A (*SDHA*), and ribosomal protein L4 (*RPL4*). There were ten genes of interest: intercellular Adhesion Molecule 1 (*ICAM1*), tumor necrosis factor-α (*TNF-*α), nuclear factor kappa-light-chain-enhancer of activated B cells (*NFKB1*), interleukin 1 β (*IL1*β), interleukin 8 (*IL8*), interleukin 18 (*IL18*), interleukin 10 (*IL10*), signal transducer and activator of transcription 3 (*STAT3*), heat shock protein 90 (*HSP90*), interleukin-1 receptor antagonist protein (*IL1RN*). Primer sequences are reported in Table 2.

**Table 2:** Primer sequence for reference genes and genes of interest and qPCR efficiency.

**Gene symbol**	**Primers 5^**′**^-3^**′**^ forward, reverse**	**Gene ID**	**Amplicon bp**	**E %**	**R^**2**^**
**Reference genes**					
*ACTB*	CCTGAACCCCAAGGCCAACCG GGCGTACAGGGACAGCACGG	XM_003357928	106	97.8	0.999
*B2M*	CAACCACTTTTCACACCGCTCCAGT ACCGCATCCAGGCCAGACAGT	NM_213978	91	99.4	1.000
*GAPDH*	ACACTCACTCTTCTACCTTTG CAAATTCATTGTCGTACCAG	DQ_845173	90	102.0	1.000
*HPRT*	GCAGCCCCAGCGTCGTGATTAG AGCAAGCCGTTCAGTCCTGTCC	NM_001032376	140	99.0	1.000
*SDHA*	GTGGTGGTTGGTGCCGGAGG GCCTCCCTGGGCTGCAACAG	XM_003362140	126	101.2	0.998
*RPL4*	CCGCGCACCACGCAAGAAGA CCTGGCCTGGCGAAGAATGGT	DQ_845176	130	97.0	1.000
**Genes of interest**					
*IL1β*	ACCCGCCAAGGAAGTGATGGC GGTCCAGGTTTTGGGTGCAGCA	NM_214055	104	96.0	0.999
*ICAM1*	GCTCACCCACCGCTGCATGT CGGAACCGGGCATCTGTCGG	NM_213816	123	97.6	0.998
*IL18*	ATGGCTGCTGAACCGGAAGAC ACACGGCTTGATGTCCCTGGT	NM_213997	193	99.4	0.999
*IL8*	GGTCTGCCTGGACCCCAAGGA TGGGAGCCACGGAGAATGGGT	NM_213867	124	97.2	1.000
*IL10*	GAGCTGCCTTCGGCCCAGTGA ACAAGGCTTGGCAACCCAGGTA	NM_214041	117	100.2	1.000
*TNF*	GCCCGTCGCCCACGTTGTAG CTTCACGCCGTTGGCCAGGA	NM_214022	94	102.6	1.000
*IL1RN*	GTGTCCTGTTGTTGCATGGTC AGGTCTCTTTCCCAAGGGGT	NM_214262	123	97.9	0.998
*HSP90*	TCCCCAGACGCACGCCAACA AGGGGTGGCATCTCCTCCGT	NM_213973	117	98	1.000
*NFKB1*	CACTCGCTGCCCTTCTCGCC AAAGGACGTCTCCACGCCGC	NM_001048232	93	99.0	1.000
*STAT3*	CCGGCCCATGCTGGAGGAGA GCCGGTCAGGGTGCATAGGC	NM_001044580	104	99.8	1.000

*Amplicon bp, amplicon length, base pairs; E, efficiency of assay; R^2^, correlation coefficient*.

Whenever possible, primers were designed on different exons or at an exon–exon junction to minimize inaccuracies due to possible residual genomic DNA contamination. Amplicon lengths were optimized to 90/193 bp to ensure optimal amplification efficiency. The specificity of amplification was confirmed by sequencing. For each pair of primers, a preliminary qRT-PCR was performed and the efficiency (E) was assessed by assigning slope values and correlation coefficients (R^2^) reported in Table 2.

Reactions were carried out in a final volume of 20 μl using 5 μl of a 10-fold diluted cDNA template, FastStart SYBR Green Master mix (Roche Applied Science, Penzberg, Germany), ROX fluorochrome as an internal check on an MX3000P instrument (Stratagene, Santa Clara, CA, USA) following the same conditions for all primer pairs: 95 °C for 10 min, 40 cycles of 95 °C for 30 s, 58 °C for 30 s. Fluorescence data were collected at the end of the extension step. Each reaction was run in triplicate and, at the end of each run, the melting curve was determined in the range of 58–95 °C with a temperature increment of 0.01 °C/s. For analysis, the raw Cq values were imported to the GenEx Pro (version 6) software package (MultiD, Göteborg, Sweden). The stability of the six RGs was tested using geNorm (46) and the NormFinder algorithms (47) included in the GenEx Pro software (MultiD, Göteborg, Sweden).

### Analysis of RT-qPCR Data

The raw Cq values were analyzed at three time-points: T0, T1, and T2. During the pre-processing step, data were corrected for the E values of primers (Table 2), normalized for the two most stable RGs (*HPRT, GAPDH*), quantified relative to the maximum Cq value, and converted in log2.

### Statistical Analysis

The gene expression data from the GenEx Pro software (MultiD, Göteborg, Sweden) were analyzed using the GLM procedure of SAS (JMP 9; SAS Institute Inc., Cary, NC, 2010). Before the analysis, data distributions for each gene were checked for normality using the Shapiro–Wilk test. A mixed design ANOVA, where the rearing system (two levels: IN and OUT) and diet (two levels: CTRL and ORE) were included as between-subjects factors, time as a repeated factor, and T0 as covariate, was used. Except for diet^*^rearing system, all other tested interactions between the main factors were not significant (*p* > 0.05) and were removed from the model. The Tukey–Kramer adjusted *t*-test was applied to assess the difference between means. We considered statistically significant differences when *p*-value was lower than 0.05. Data were expressed as least squares (LS) means ± standard error (SE).

## Results

During the whole experimental period, the animals underwent regular veterinary checks and no pathologies were observed.

### Selection of Optimal RGs

To evaluate the most stable genes to be used for normalization purposes, six potential RGs (*ACTB, B2M, GAPDH, HPRT, RPL4*, and *SDHA*) were tested in 48 samples, randomly chosen at each time point (T0, T1, T2), at the farming system (IN, OUT), and at diet treatment (CTRL, ORE). The six genes were analyzed by qRT-PCR to estimate their efficiency according to Cappelli et al. (48) (Table 3). The Cq value for the six RGs tested ranged between 18.0 (*ACTB*) and 25.5 (*HPRT*). Tables 3, 4 show the ranking of the six candidate RGs, based on their stability values calculated using geNorm and NormFinder, respectively.

**Table 3:** Candidate reference genes ranking according to geNorm.

**Rank**	**Gene symbol**	**M-value**
$\frac{1}{2}$	*GAPDH/HPRT*	0.223
3	*B2M*	0.264
4	*SDHA*	0.319
5	*ACTB*	0.357
6	*RPL4*	0.419

*M-value, average pair-wise variation of each gene against all others*.

**Table 4:** Candidate reference genes ranking according to NormFinder.

**Rank**	**Gene symbol**	**SD**	**Accumulated SD**
1	*HPRT*	0.164	0.164
2	*B2M*	0.178	0.121
3	*GAPDH*	0.191	0.103
4	*SDHA*	0.335	0.114
5	*ACTB*	0.358	0.116
6	*RPL4*	0.494	0.127

*SD, standard deviation*.

The results obtained using geNorm and NormFinder showed that three RGs (*HPRT, GAPDH*, and *B2M*) were always classified among the top positions in the stability rankings produced by both algorithms.

Overall, on the basis of the expression stability values collected in this study, an optimal normalization factor for qRT-PCR data could be calculated using the two most stable genes, *HPRT* and *GAPDH*.

### Gene Expression Analysis

#### Effect of Dietary Treatment (CTRL vs. ORE)

When comparing the effect of the dietary treatment on gene expression, only *HSP90* was downregulated in pigs fed with the ORE diet (*P* < 0.01). Results are shown in Table 5.

**Table 5:** Diet, farming system, and time effects on gene expression.

**Items**	**Dietary treatment**		**SEM**	***P*-value**	**Rearing system**		**SEM**	***P*-value**	**Sampling time**		**SEM**	***P*-value**
	**CTRL**	**ORE**			**IN**	**OUT**			**T1**	**T2**		
*HSP90*	1.117	0.857	0.069	0.010	0.807	1.167	0.069	0.001	0.838	1.135	0.070	0.003
*ICAM1*	1.281	1.088	0.105	0.202	1.118	1.240	0.105	0.452	1.056	1.313	0.088	0.009
*IL8*	5.276	4.907	0.283	0.356	5.750	4.433	0.283	0.002	5.631	4.551	0.288	0.009
*IL18*	1.823	1.492	0.128	0.079	1.645	1.669	0.128	0.898	1.596	1.718	0.109	0.299
*TNF*	1.280	1.335	0.092	0.679	1.164	1.452	0.092	0.036	1.205	1.410	0.076	0.011
*IL10*	1.703	1.522	0.143	0.379	1.713	1.512	0.143	0.330	1.810	1.416	0.132	0.023
*IL1RN*	1.388	1.437	0.111	0.754	1.736	1.089	0.111	0.003	1.216	1.608	0.093	0.001
*NFKB1*	1.427	1.265	0.062	0.073	0.990	1.702	0.063	<0.001	1.182	1.510	0.056	<0.001
*IL1β*	1.711	1.653	0.102	0.694	1.465	1.897	0.101	0.004	1.681	1.680	0.103	0.997
*STAT3*	0.919	0.852	0.060	0.435	0.682	1.088	0.060	<0.001	0.607	1.163	0.061	<0.001

*CTRL, control diet; ORE, oregano essential oil dietary supplementation; IN, indoor rearing system; OUT, outdoor rearing system; T1, 120 days; T2, 190 days; SEM, standard error of the mean*.

#### Effect of Rearing System (IN vs. OUT)

The expression of seven genes changed between pigs reared in the IN vs. OUT systems (Table 5). The expression of *IL8* and *IL1RN* was upregulated in pigs reared in the IN system (*p* < 0.01), whereas the expression of *HSP90, TNF, NFKB1, IL1*β, and *STAT3* was upregulated in pigs reared in the OUT system (*p* < 0.05).

#### Effect of Sampling Time

Comparison at T0, before the administration of the experimental diets, showed no significant differences (*p* > 0.05) between groups for all the tested genes before the administration of the experimental diets.

With the exception of *IL18* and *IL1*β, all genes had a differential expression in T1 vs. T2 (Table 5). The expression of *IL8* and *IL10* was higher in T1, whereas the expression of *HSP90, ICAM-1, TNF, IL1RN, NFKB1*, and *STAT3* was higher in T2 (*p* < 0.05).

#### Effect of the Interaction Between Rearing System (IN vs. OUT) and Diet (CTRL vs. ORE)

As shown in Table 6, the expression of *HSP90, NFKB1*, and *STAT3* was significantly downregulated in the OUT reared pigs fed with the ORE diet while, in the IN pigs, these genes were basically less activated and not modulated by the dietary treatment (rearing system × diet: *p* < 0.05, *p* < 0.001, and *p* < 0.01, respectively).

**Table 6:** Rearing system and dietary treatment interaction effects on gene expression.

**Items**	**IN**		**OUT**		**SEM**	***P*-value**
	**CTRL**	**ORE**	**CTRL**	**ORE**		
*HSP90*	0.818^b^	0.796^b^	1.416^a^	0.918^b^	0.099	0.017
*ICAM1*	1.243	0.868	1.318	1.307	0.149	0.422
*IL8*	5.987	5.511	4.565	4.301	0.407	0.791
*IL18*	1.748	1.543	1.898	1.440	0.184	0.493
*TNF*	1.064	1.263	1.496	1.407	0.132	0.280
*IL10*	1.870	1.556	1.536	1.488	0.205	0.516
*IL1RN*	1.733	1.738	1.042	1.136	0.158	0.776
*NFKB1*	1.063^c^	0.917^c^	1.938^a^	1.466^b^	0.088	0.001
*IL1β*	1.287	1.644	2.076	1.718	0.142	0.118
*STAT3*	0.781^bc^	0.583^c^	1.254^a^	0.922^b^	0.087	0.003

*IN, indoor rearing system; OUT, outdoor rearing system; CTRL, control diet; ORE, oregano essential oil dietary supplementation; SEM, standard error of the mean*.

a, b, c *Different superscript letters in the same row mean significant differences at p < 0.05*.

## Discussion

Our molecular data collectively support the hypothesis that the interaction between the rearing system (IN vs. OUT) and the dietary supplementation with the ORE influences a group of inflammatory response genes in pigs. Hereafter, we will begin by discussing this interaction; then, we will evaluate the main effects exerted by the rearing system and time of sampling, which is in turn associated with the climatic changes of the year.

### Effect of Interaction Between Rearing System and Diet

Our data showed a significant interaction between dietary treatment and the rearing system for a number of genes (Table 6). This suggests that the oregano bioactive compounds act in modulating the inflammatory signaling involved in response to stressful stimuli, only when these are in action.

NFKB1 is a transcription factor and also a signaling hub in leukocytes (49, 50); indeed, the transcript of this protein was upregulated in the outdoor conditions when compared to the indoor conditions (Table 5). The lower expression of *NFKB1* in the OUT pigs fed with the ORE diet indicates that oregano could be effective in mitigating pro-inflammatory pathways by means of a leukocyte response driven by the NFKB1 transcription. Our data confirm previous findings on the role of oregano as an inhibitor of NFKB1 (37, 51). Indeed, oregano exhibited a significant ability to inhibit the NFKB1 activity induced by lipopolysaccharide in a monocyte cell line *in vitro* (51). Moreover, the expression of *NFKB1* in the small intestine of broilers challenged with lipopolysaccharide was reduced after oral administration of carvacrol (52), one of the major components of ORE (Table 1) (53, 54). Carvacrol is an immunomodulant and an anti-inflammatory molecule known to reduce protein concentration as well as the gene expression modulation of interleukins (55, 56).

In addition to this well-established pathway, oregano was shown to modulate microbial species in the intestine (37) and reduce pathogen contamination (38). It is known that the modulation of gastrointestinal microbiota can influence systemic immune responses (57). It has also been demonstrated that oregano has positive effects on enterocytes by accelerating their renewal rate, improves their capacity for nutrient absorption, and promotes integrity of the intestinal barrier in pigs (37). Enterocytes, in turn, are in close relationship with the intestinal immune system and constitute a functional barrier to dietary and microbial antigens. For these reasons, we cannot exclude that, in our experimental conditions, oregano has indirectly affected gut mucosa functionality and digestion in a beneficial manner.

Finally, it should be stressed that NFKB1 and STAT3 are transcription factors known to modulate the heat shock protein (HSPs) expression during the immune response (58–60). These proteins are chaperones in protein folding, activation, and assembly and prevent tissue damage due to stress stimuli. It is known that HSPs are upregulated and modulate inflammatory pathways in pigs under inflammatory conditions (61), especially heat stress (62–64). In our experiment, samples at T1 and T2 were collected at the end of summer and in full winter, respectively, in an undoubtedly stressful situation for the animals. The modulating influence exerted by the ORE diet was observed under the OUT conditions, where the genes of the response to thermal stress were more actively upregulated (Table 5).

STAT3 encodes a protein which activates in response to cytokines, especially IL10 (65). STAT3 protein translocate into the cell nucleus and acts in blocking the release of pro-inflammatory cytokines (66). The upregulation of this gene under OUT conditions (Table 5) may depend on the need to moderate the inflammatory process due to chronic stressful stimuli, such as exercise or climate adaptation, a mechanism already known to protect the organism from activated macrophage “overshooting,” with potential tissue damage *via* the IL10/STAT3 pathway (67, 68). In our data, *IL10* was not significantly modulated by the rearing system, whereas *IL8* was downregulated; we can hypothesize that, at T1, STAT3 activator transcripts IL10 had already returned to their baseline transcription after inducing the transcription of STAT3 (Table 5).

Our data show that, when administered under OUT conditions, the ORE diet led to a significant decrease in the STAT3 expression, and its inductor transcripts (pro-inflammatory cytokines NFKB1, IL8, IL18, and anti-inflammatory IL10) were not modulated in these groups of animals (Table 6).

Moreover, in the same group of animals, the ORE diet prevented IL1β from increasing. IL1β is a prototypic proinflammatory cytokine, which exerts effects on a variety of cells and plays a key role in acute and chronic inflammation. IL1β has important homeostatic functions in the normal organism. However, the overproduction of IL1β is implicated in the pathophysiological changes, which occur during different disease states (69).

### Effect of Rearing System (IN vs. OUT)

It is known that various stressors involved in the OUT rearing condition can alter the physiology of an intestinal barrier and can affect the production of pro-inflammatory cytokines and chemokines in pigs (70, 71). These factors, coupled with changes in the diet associated to the OUT environment, have the potential to modulate the microbiome at the mucosal level and, consequently, at the intestinal immune response (70). Early life, especially the time of weaning, is crucial in establishing microbiota and the immune system in pigs (57). Indoor or outdoor environments can have a differential impact on these phenomena (72). For instance, an increased physical complexity of rearing environment seems to generate a less diverse microbial community when compared to indoor-raised pigs (73).

Differences in intestinal microbial population can be related to transcriptional modulations in a number of genes. When comparing two groups of pigs, either reared under outdoor or indoor conditions, large differences in the ileal mucosa-adherent microorganisms were found (72). These differences were associated to an increased expression of major histocompatibility complex class I (MCHI) and chemokines that indicate the presence of an immune-activated gut microenvironment (72).

Under our experimental conditions, the majority of transcripts, which refer to a general inflammatory status (*TNF, HSP90, NFKB1, IL1*β, and *STAT3*), showed an upregulation in the OUT environment, whereas transcripts encoding *IL8* [inhibited by STAT3 (74)] and *IL1RN* were downregulated as shown in Table 5. This pattern is possibly related to a stimulation of the immune system by the stressful stimuli present in outdoor rearing, such as exercise and harsh climatic conditions, which can activate the inflammatory response pathways. As previously detailed, the environmental conditions of the OUT pigs have most likely affected the level of stress in both hot and cold seasons of the year (cold winds and rain in winter, solar irradiation in summer, the absence of straw bedding, physical exercise, and the presence of a thick layer of mud in rainy periods).

Both *IL1*β and *TNF* transcripts, upregulated in the OUT conditions, transcribe well-known pro-inflammatory cytokines released from various immune cells (mainly monocytes and macrophages), and their production suggests the onset of an inflammatory response (75–78). The circulating levels of TNF have also been indicated as a marker of inflammation in pigs challenged with common pathogens, such as *Escherichia coli* (79–82). However, in our experimental conditions, no pathologies were found. The upregulation of these two cytokines in our study might reflect the exposure to thermal stress and to the environmental stressors, like physical exercise, that exacerbate this response.

In addition, pro-inflammatory cytokine stimuli, such as TNF-α and IL-1 (49, 50, 83–85), can activate NFKB1; its roles as a transcription factor and a signaling hub in leukocytes were previously described. Together with NFKB1, we have discussed how the ORE diet was able to modulate the expression of two other major genes (*STAT3* and *HSP90*) involved in the inflammatory process and markedly (*p* < 0.001) upregulated under the OUT conditions (Table 5).

In contrast, *IL1RN* transcripts were downregulated under the OUT conditions. The *IL1RN* gene encodes IL1Ra, a protein that binds non-productively to the outer cell surface domain of the IL-1R. IL1Ra is secreted by various types of cells, including immune cells, and is a natural inhibitor of the pro-inflammatory effect of IL1β. The balance between IL1β and IL1RN determines whether the initial pro-inflammatory response will persist or regress (86). The downregulation of this gene, together with the upregulation of IL1β, contributes to explain the inflammatory response recorded under the OUT conditions.

### Effect of Sampling Time

The expression of most tested genes was affected by time: six genes out of the eight were upregulated in T2 as compared to T1 (Table 5).

Since the pigs were acclimated to the environment prior to the study, this suggests that, during the experiment, the environment changed over time. Indeed, blood samples were taken in September (T1), after hot summer weather conditions, and in January (T2), in the middle of winter with opposite weather conditions.

The inflammatory response is likely associated with the upregulation of *TNF, HSP90, IL8, NFKB1*, and *ICAM1*. The *ICAM1* gene encodes a cell surface glycoprotein, typically expressed on cells of the immune system and binding integrins to activate the immune response. It is usually upregulated during inflammatory conditions (87) in response to the pro-inflammatory stimuli of cytokine, especially IL8 and TNF (88, 89).

In contrast, it is likely that the observed increase of IL1RN transcription at T2 was intended to antagonize the pro-inflammatory cytokine stimuli (Table 5).

Taken all together, these results lead to the same direction as other encouraging number of studies regarding the molecular mechanism underlying the beneficial effects of dietary oregano were administered under stressful conditions. They report a decrease in the inflammatory cytokine levels in the pig jejunum, following transport stress (32), an inhibition of the NFKB1 transcription factor on PBMCs *in vitro* (36, 51), and a decrease in the mortality rate. They also show an acceleration in the recovery of a gain in body weight, a reduction in tissue damage, and a decrease in the mRNA levels of pro-inflammatory cytokines (90).

## Conclusion

Our overall observations seem to suggest that the ORE diet can have a mitigation effect on the inflammation state induced by non-infective stress. Indeed, the observed interaction between diet and the farming systems indicates that the beneficial effects of bioactive compounds contained in the OREs are likely to exert their activity when the animals are exposed to stressful stimuli. It is known that stressors involved in outdoor rearing conditions can have an impact on the gut ecosystem and favor inflammatory processes. These factors, often associated with changes in the diet, can influence the intestinal microbiota and alter immune systems at both systemic and local levels.

We can speculate that the ORE dietary supplement can be an acceptable strategy to help pigs tolerate the stress related to harsh, outdoor, rearing conditions. Indeed, these findings based on the molecular data are confirmed by growth performance results reported by our research group in another study, where the same animal cohort was used (11).

In view of the consumer positive attitudes toward extensive rearing systems and animal welfare, further studies should be encouraged to better understand the effects of outdoor environmental constraints and the possible beneficial role of phytoderivatives in livestock diets.

## Data Availability Statement

The original contributions presented in the study are included in the article/supplementary materials, further inquiries can be directed to the corresponding author/s.

## Ethics Statement

The animal study was reviewed and approved by Council of the Pathology, Diagnostics and Veterinary Clinics Department, University of Perugia (Department Council board minutes no. 8 of 28 June 2010). All blood samples were collected during 2012–2013, before the implementation of Italian Legislation Decree n. 26/2014 which requires a specific authorization of Ministry of Health.

## Author Contributions

MT-M, KC, and GA conceived the study and participated in its design. KC choose the research methodology. SC, AV-S, and MT-M carried out the formal analysis. KC, GA, MS, and LM performed the investigation. AV-S, MT-M, and LM provided the funding acquisition. SC, GA, and MT-M performed the data curation. KC, MT-M, and MS participated in writing-original draft preparation. AV-S, SC, and GA participated in writing-review and editing. AV-S and MT-M supervised the research. All authors have read and contributed to the final manuscript and approved the submitted version.

## Conflict of Interest

The authors declare that the research was conducted in the absence of any commercial or financial relationships that could be construed as a potential conflict of interest.
